# Effect of Sucrose on the Rheology and 3D Printability of Pregelatinized Rice Flour Paste

**DOI:** 10.3390/foods14071107

**Published:** 2025-03-23

**Authors:** Dongju Lee, Mohammed I. Saleh, Youngseung Lee

**Affiliations:** 1 Department of Food Science and Nutrition, Dankook University, Cheonan-si 31116, Republic of Korea; dongju042027@naver.com; 2 Department of Nutrition and Food Technology, School of Agriculture, The University of Jordan, Amman 11942, Jordan; misaleh@ju.edu.jo

**Keywords:** sucrose, pregelatinized rice, 3D printing

## Abstract

Although sucrose plays a crucial role in influencing the rheological and textural properties of sucrose–starch-based blends, its effects on the 3D printability and rheological behavior of α-rice flour paste remain largely unexplored. This study aimed to investigate the impact of varying sucrose concentrations (0, 1, 2, 3, and 4% *w*/*w*) on the printability and rheological properties of α-rice flour paste. Printability was evaluated using a 3D food printer, while rheological properties were analyzed using a rheometer. All the samples exhibited shear-thinning behavior. As the sucrose concentration increased, both the maximum storage modulus (G′_Max_) and yield stress (τy) decreased significantly, while the printing percentage error and deformation factor increased. A strong negative linear correlation was observed between G′_Max_, τy, and the printing percentage error (R^2^ = 0.94, 0.95), whereas the deformation factor exhibited a negative quadratic correlation (R^2^ = 0.99, 0.94). These results indicate that a decrease in α-rice flour concentration combined with sucrose addition weakens viscoelastic properties, resulting in lower structural stability and greater deformation. This study confirms the role of α-rice flour in enhancing starch’s physical and functional properties and provides fundamental data for optimizing 3D printing with α-rice flour–sucrose paste.

## 1. Introduction

Three-dimensional (3D) printing, or additive manufacturing, is a technology that constructs structures layer by layer using computer-aided designs [1]. This technology is widely utilized across various industries, including aerospace, automotive, healthcare, and food sectors. In particular, 3D food printing has gained significant attention due to its ability to meet diverse demands related to nutrition, color, texture, flavor, aroma, and shape, as well as its potential for personalized nutrition and the precise fabrication of food structures [2]. This technology, with its capability to precisely fabricate complex food structures, offers diverse industrial applications, including personalized nutrition delivery, texture modification, and functional food development. Furthermore, by integrating with digital cooking technology, it enables the efficient production of specialized nutritional foods and alternative protein-based products while optimizing manufacturing processes by reducing time and labor requirements, establishing itself as an innovative food manufacturing approach [3,4]. Additionally, it enables the production of creative and innovative food products that are challenging to achieve with conventional manufacturing methods, positioning it as an eco-friendly, cutting-edge technology [2].

Rice (*Oryza sativa* L.) is one of the most widely consumed grains globally, known for its excellent energy supply [5]. However, its low protein and gluten content limit its ability to form cohesive network structures, impart viscoelastic properties, and ensure stability, thereby reducing its suitability as a material for 3D food printing [6]. These limitations primarily stem from the composition and structural characteristics of rice starch. Rice starch consists mainly of amylose and amylopectin, with amylopectin being the dominant component [7]. Amylopectin has a branched structure with a backbone of α-(1-4)-linked glucose units and branch points formed by α-(1-6) linkages approximately every 20–30 glucose units [7]. This branched architecture facilitates cross-linking within starch granules, significantly influencing the rheological properties of starch-based mixtures. Furthermore, the native starch in rice possesses a complex semi-crystalline structure, rendering it water-insoluble in water at room temperature, which restricts its industrial applications [8]. To overcome these limitations and expand its utilization in the food industry, modifying the structure of starch is of significance for processing rice into an ink material suitable for 3D printing [9]. Starch modification can be achieved through chemical, physical, and enzymatic methods. Notably, pregelatinized starch (PGS), produced via physical modification is regarded as a clean-label processing method, as it does not involve the use of chemical additives or synthetic reagents. Clean-label products are typically defined as those perceived to be natural, minimally processed, and devoid of artificial additives [10].

It enhances water absorption and swelling in cold water, thereby improving viscosity and providing superior thickening properties [9]. PGS contributes to overcoming the limitations of native starch by reinforcing the network formed by natural starch granules and enhancing rheological properties [11]. Further, PGS promotes water dispersion and absorption, forms stable gels, and improves viscoelasticity and structural stability, making it a suitable material for 3D printing applications [12].

To broaden the application of PGS in the food industry, various food additives, such as sugars, salts, and organic acids, are employed to modify and improve starch properties [13]. Among these, hydrocolloids regulate interactions between starch molecules, increasing viscosity and enhancing structural stability, while salts modify the electrostatic interactions between starch and water, influencing gelatinization temperature and extrusion properties [14]. In contrast, sucrose has been reported to influence the gelatinization, retrogradation, rheological, and textural properties of starch depending on the type and concentration of starch [8]. This is attributed to sucrose’s ability to disrupt hydrogen bonding between starch molecules or modulate the availability of water molecules, thereby affecting starch swelling, viscosity, flow behavior, and structural stability [15]. Additionally, sucrose, as a highly adhesive food component, modulates the adsorption and aggregation behavior of starch-based paste in 3D printing inks, influencing extrudability and interlayer adhesion [16]. These properties play a critical role in the 3D printing process, as variations in sucrose concentration alter the rheological characteristics of starch-based materials, significantly impacting printing accuracy and structural stability.

Rheological behavior is a key factor determining the printability of 3D-printed materials, providing critical insights into material behavior throughout the printing process [17,18]. In particular, the suitability and quality of food pastes for 3D printing largely depend on their rheological properties. Recent studies have identified ease of extrusion and the ability to maintain structural stability after deposition as essential criteria for evaluating 3D printability [1]. Materials that meet these criteria must be easily extrudable through the nozzle while maintaining structural integrity during and after the printing process [1]. Additionally, since materials experience high shear stress during nozzle extrusion, possessing an appropriate yield stress is crucial to ensure smooth extrusion flow [19].

Previous studies on the effects of food additives on starch properties include investigations on the effect of sodium chloride solution on the quality of 3D-printed samples molded using wheat starch gel [20]. the effects of sugars and sugar alcohols on the pasting and granular swelling of wheat starch [21], the effect of NaCl on the formation of starch-lipid complexes [22], the effect of NaCl addition on alcohol–alkali-treated waxy rice starch: Structural and physicochemical functionality [23], and the effects of processing and additives on starch physicochemical and digestibility properties [24]. However, correlation studies analyzing the effects of sucrose, as a food additive, on the printability and rheological properties of PGS are still limited.

Therefore, this study aims to evaluate the effects of varying sucrose concentrations (0, 1, 2, 3, and 4% *w*/*w*) on the printability and rheological properties of α-rice flour including PGS using a food model system, and to analyze the correlations between these properties. The findings of this study are expected to elucidate the influence of sucrose on PGS-based 3D printing and provide fundamental data for the formulation of optimized ink compositions tailored for PGS-based 3D printing processes.

## 2. Materials and Methods

### 2.1. Materials

Commercially available α-rice flour (100% domestic rice, Sejun F&B Co., Ltd., Gangwon-do, Republic of Korea) and white granulated sugar (CJ CheilJedang Co., Ltd., Seoul, Republic of Korea) were purchased from a local supermarket and used as experimental samples. Distilled water (Milli-Q Direct 16 system, Merck, Darmstadt, Germany) was utilized throughout the experiments.

### 2.2. Preparation of α-Rice Flour–Sucrose Paste

The α-rice flour–sucrose paste was prepared according to the formulations presented in Table 1. The pastes were produced with varying sucrose concentrations (1, 2, 3, and 4% *w*/*w*), while the mixture without sucrose served as the control. Sucrose was dissolved in distilled water (~25 °C) at concentrations of 1, 2, 3, and 4% (*w*/*w*) and stirred for 5 min at room temperature (~25 °C) using a magnetic stirrer. This temperature was chosen based on the ability of pregelatinized rice flour to form viscosity at room temperature without additional heating, ensuring consistency with standard application conditions and practical reproducibility. Subsequently, α-rice flour, sieved through an 80-mesh screen, was added and manually mixed for 5 min to obtain the α-rice flour–sucrose paste. Five minutes was sufficient to ensure a uniform mixture of α-rice flour–sucrose paste, as confirmed by preliminary tests. The uniformity of the mixture was evaluated through visual inspection to confirm the homogeneous dispersion of α-rice flour within the sucrose solution. Samples were designated as S0, S1, S2, S3, and S4 based on sucrose concentration.

The formulation was selected based on preliminary experiments to determine optimal rheological properties for 3D printing. Initially, varying ratios of α-rice flour to distilled water without sucrose were tested. A concentration of 30–32% (15–16 g) α-rice flour resulted in overly fluid pastes, while 36–37% (18–18.5 g) was too viscous for 3D printing. The optimal printability was observed at 35% (17.5 g) α-rice flour, which was used as the control formulation. Sucrose concentrations above 5% (2.5 g) led to excessive fluidity, while concentrations around 2% (1 g) increased viscosity. Thus, the experimental range for sucrose concentration was set between 0–4% (0–2 g).

### 2.3. Rheological Measurement

The rheological properties of the α-rice flour–sucrose paste were analyzed using a rheometer (HAAKE Viscotester iQ, Thermo Scientific, Dreieich, Germany) equipped with a cone-and-plate geometry (diameter: 60 mm, gap: 1 mm) at 20 °C. Measurements were conducted at a fixed frequency of 1 Hz, with shear stress increased logarithmically from 10 to 1000 Pa. Approximately 1.25 mL of the paste was placed on the rheometer plate and uniformly compressed to a 1 mm thickness.

**Figure 1:**
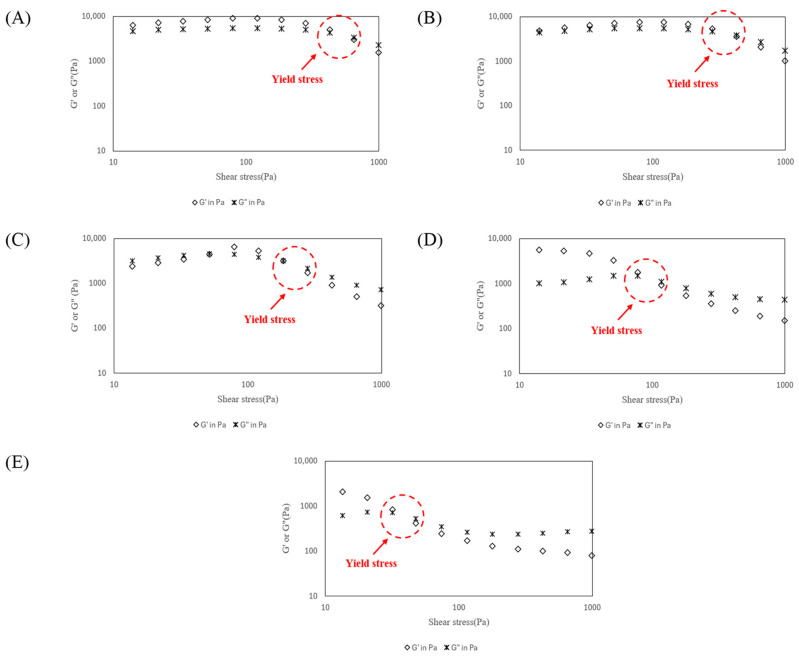
Representative oscillation amplitude sweeps for (**A**) S0, (**B**) S1, (**C**) S2, (**D**) S3, and (**E**) S4. The τy was determined as the intersection G′ equals G″.

The maximum storage modulus (G′_Max_) and loss modulus (G″_Max_) within the linear viscoelastic region were evaluated and compared with printability data. Yield stress (τy) was defined as the crossover point of G′ and G″ during oscillatory amplitude sweep measurements, following the method of Gholamipour-Shirazi et al. [25] (Figure 1). This approach focused on comparing the relative τy among samples rather than determining absolute values. All the rheological measurements were performed in triplicate.

### 2.4. 3D Printing Process

**Figure 2:**
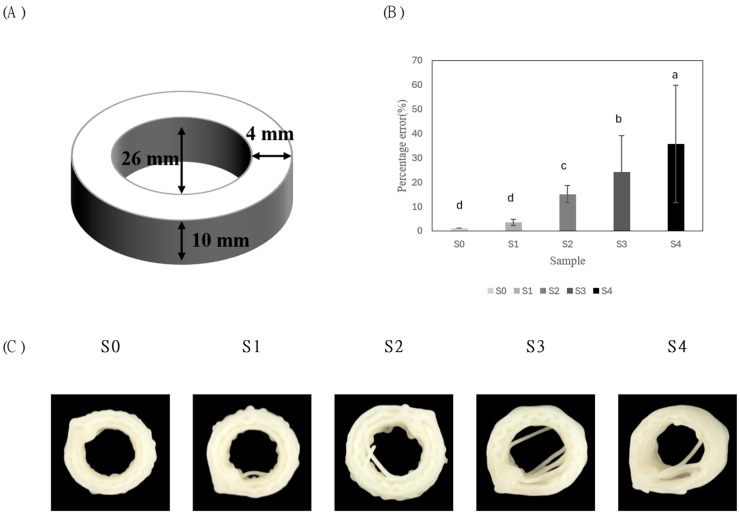
(**A**) Designed 10 mm height hollow cylinder with a 4 mm wall thickness and 26 mm inside diameter. (**B**) Average printing percentage error of printed structures. Values with different lowercase letters on the samples are statistically different (*p* < 0.05). (**C**) Comparative top view of the printed structures.

**Figure 3:**
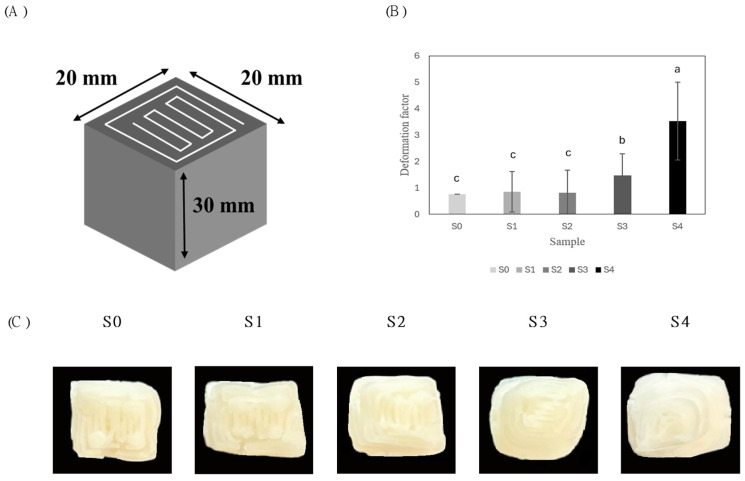
(**A**) Designed a 20 mm × 20 mm × 30 mm Solid cuboid. (**B**) Average deformation factor of printed structures. Values with different lowercase letters on the samples are statistically different (*p* < 0.05). (**C**) Comparative top view of the printed structures.

The 3D printing of the α-rice flour–sucrose pastes with varying sucrose concentrations was conducted using a 3D food printer (YOLILO Co., Ltd., Seoul, Republic of Korea). The paste was loaded into 50 mL syringes, and the 3D designs (.stl file format) included a hollow cylinder structure (26 mm diameter, 10 mm height, as shown in Figure 2) and a cuboid structure (20 mm length/width, 30 mm height, as shown in Figure 3).

The printing parameters were configured using Repetier-Host 2.0.5 as follows: printing temperature of 25 °C, nozzle diameter of 1.1 mm, layer height of 0.8 mm, first layer height of 1.7 mm, 100% infill with a linear pattern, and nozzle speed of 30 mm/s. In 3D printing, it has been reported that setting the layer height equal to the nozzle diameter or at approximately 80% of the nozzle diameter improves the shape retention and adhesion of the deposited filaments [26]. Therefore, in this study, the nozzle diameter was set to 1.1 mm and the layer height to 0.8 mm to ensure structural stability during the printing process.

### 2.5. Printability Evaluation

Printability was evaluated based on the method described by Maldonado-Rosas et al. [3], comparing the designed area of the 3D model with the actual printed structure or comparing the top and bottom surface areas of the printed object. After printing, the structures were stored at room temperature for 24 h before imaging with a ruler for scale. The images were analyzed using ImageJ software (version 1.54) to measure the internal area of the hollow cylinder structures and the top and bottom areas of the cuboid structures. The images were scaled and converted to 8 bit format for analysis.

The printing percentage error was calculated using the hollow cylinder structure (diameter 26 mm, height 10 mm). The internal area was measured three times and compared with the design specifications. The printing percentage error was calculated using Equation (1):

(1) $$P_{error}=\frac{\left(A_{measured}-A_{designed}\right)}{A_{designed}}\times 100.$$

In Equation (1), $P_{error}$ represents the printing percentage error, $A_{masured}$ denotes the average measured internal area, and $A_{measured}$ refers to designed area.

The deformation factor was calculated using the cuboid structure (length, width, and height = 20, 20, and 30 mm, respectively). It was estimated by comparing the top and bottom surface areas of the cuboid structure, with the average value from three repeated measurements calculated using Equation (2).

(2) $$Deformation\;factor=\frac{A_{Bottom}}{A_{top}}.$$

In Equation (2), $A_{Bottom}$ represents the area at the bottom of the structure, while $A_{top}$ denotes the area at the top.

### 2.6. Statistical Analysis

All experimental results were expressed as mean ± standard deviation. Data were analyzed using a one-way analysis of variance (ANOVA) with XLSTAT software (version 2024 for Windows, Addinsoft, Inc., Paris, France). Significant differences for the measured parameters between samples were determined using Tukey’s post-hoc test (*p* < 0.05). In this study, ANOVA was applied to analyze significant variables related to the rheological properties and printability of 3D printing. Fernández-Cervantes et al. [27] was referenced to incorporate the ANOVA and Tukey HSD test approach used in the evaluation of the mechanical properties of 3D-printed scaffolds into our data analysis. Detailed ANOVA results for G′_Max_, G″_Max_, τy, printing percentage error, and deformation factor are presented in Supplementary Tables S1–S5.

## 3. Results and Discussion

### 3.1. Rheological Behavior Evaluation

The changes in storage modulus (G′), loss modulus (G″), and τy of the α-rice flour–sucrose paste as a function of shear stress are presented in Figure 1 and Table 2. G′ represents the elastic behavior of the material, while G″ indicates its viscous behavior. τy is a critical factor for ink extrudability, reflecting the minimum force required for the ink to start flowing [4]. To ensure smooth flow through the nozzle and maintain the structural integrity of the printed object after deposition, G′ and τy play essential roles [4].

As shown in Figure 1, the observed reduction in τy of the products with increasing sucrose concentration indicates the significant role of sucrose in altering the material’s viscoelastic properties. This is likely due to the competition for water molecules between sucrose and starch, which disrupts starch network formation, weakens structural cohesion, and consequently enhances flowability [28]. Yield stress fluids retain solid-like properties below a critical stress (τc) but begin to flow and deform like a liquid once this threshold is exceeded, as the internal structure collapses under external force [15].

Furthermore, the decline in G′ with increasing sucrose concentration indicates a reduced ability of the paste to maintain its elastic structure essential for preserving the printed shape [15]. At the same time, shifts in G″ suggest changes in the material’s viscous response, meaning that while higher sucrose levels may improve extrusion, they could also increase the risk of structural collapse after deposition if G′ becomes too low compared to G″ [29].

From a 3D printing standpoint, the balance between these rheological properties is key. Lower τy and reduced G′ can facilitate smoother extrusion, but if the reduction is too significant, the printed structure may lack stability, leading to deformation or sagging [4]. To achieve optimal results, sucrose concentration must be carefully controlled to maintain both extrudability and structural integrity throughout the printing process.

Within the linear viscoelastic region, G′ was consistently higher than G″, indicating predominant elastic behavior over viscous flow. However, beyond the linear viscoelastic region, G′ decreased sharply due to the disruption of the starch network structure, while viscous behavior became more dominant (Figure 1). This transition was more evident with increasing sucrose concentrations, suggesting that sucrose weakens the structural stability of the starch network [30].

Table 2 presents the variations in G′_Max_, G″_Max_, and τy for α-rice flour–sucrose pastes with different sucrose concentrations (0, 1, 2, 3, and 4% *w*/*w*). Both G′_Max_ and G″_Max_ significantly decreased with increasing sucrose concentration. This reduction is attributed to the inhibition of starch–water network formation due to higher sucrose levels and the reduction in α-rice flour, which together weaken the viscoelastic properties of the paste. This decline also suggests that while sucrose enhances flowability, it simultaneously compromises the material’s ability to retain its shape post-extrusion, a key factor in 3D printing applications. Notably, S0 exhibited the highest G′_Max_ (9032.0 Pa) and G″_Max_ (5448.1 Pa), while S4 showed the lowest G′_Max_ (2212.1 Pa) and G″_Max_ (739.1 Pa). A notable trend is the steep decline in G′_Max_ and G″_Max_, where S0 (0% sucrose) exhibited the highest values, indicating the strongest gel-like behavior, while S4 (4% sucrose) had the lowest, suggesting a more fluid-like consistency. This aligns with the theory that sucrose competes with starch for water molecules, disrupting hydrogen bonding and reducing the ability of the starch granules to form a cohesive network.

Similarly, τy significantly decreased with increasing sucrose concentration. The highest τy was observed in S0 (3845.3 Pa), which decreased markedly to S4 (658.8 Pa). This might reveal a significant drop from S0 to S4, reinforcing the idea that higher sucrose concentrations facilitate flow but at the cost of mechanical strength. The hydroxyl groups in sucrose can form hydrogen bonds and other interactions with water molecules, affecting starch swelling behavior and water absorption by proteins. This alters the protein network structure, ultimately reducing the viscoelastic properties of the paste [31]. Therefore, optimizing sucrose concentration is important for enhancing the viscoelasticity of α-rice flour–sucrose paste and ensuring printability and structural stability in 3D printing processes.

### 3.2. Evaluation of the Printability

#### 3.2.1. 3D-Printed Hollow Cylinder

To evaluate the printing accuracy of the 3D structures after deposition, a hollow cylinder structure (Figure 2A) was printed. The percentage error was analyzed based on the method of Maldonado-Rosas et al. [3]. According to Figure 2B, the S4 sample exhibited the highest printing percentage error at 35.81%, which was statistically significant compared to other samples. The percentage error increased significantly with higher sucrose concentrations, rising from 1.27% in S0 to 35.81% in S4. This trend is attributed to reduced water availability due to molecular interactions between starch and sucrose within the water–starch–sucrose system, leading to weakened plasticization effects [14]. Additionally, the reduction in α-rice flour likely decreased the paste’s viscoelasticity, negatively affecting structural stability and printing precision [3]. These results suggest that decreasing α-rice flour content and increasing sucrose concentration weakens the viscoelastic properties of the paste, thereby reducing structural stability and printing accuracy. This emphasizes once again the importance of balancing the contents of starch and sugar to optimize the rheological properties and 3D printability of α-rice flour–sucrose pastes.

As shown in Figure 2C, the S4 sample exhibited collapsed internal structures, likely associated with its lowest τy. In contrast, S0 formed uniform extruded filaments, maintaining near-circular 3D shapes, indicating that sufficient τy is essential for maintaining structural stability in 3D printing [32]. Moreover, increasing α-rice flour and decreasing sucrose concentration improved the shape of internal areas and the bead morphology of printed filaments, reducing fusion between adjacent beads. α-Rice flour rapidly swells in cold water, increasing viscosity and enhancing viscoelastic properties, thus contributing to improved structural stability during printing [33]. Similarly, Maldonado-Rosas et al. [3] reported that the addition of PGS increased τy and G′, enhancing elasticity and structural stability, thereby improving shape fidelity in 3D printing.

#### 3.2.2. 3D-Printed Cuboid

To evaluate the self-supporting ability of the 3D-printed structures after deposition, a cuboid structure (Figure 3A) was printed. The deformation factor was calculated following the method described by Gholamipour-Shirazi et al. [25]. As shown in Figure 3B, the deformation factor of the α-rice flour–sucrose paste significantly increased with the rising sucrose concentration. The lowest deformation factor was observed in S0 (0.77), while S4 exhibited the highest value (3.53), indicating reduced structural stability. This increase in deformation is attributed to the weakening of the starch network with higher sucrose concentrations, which reduces the self-supporting ability of the stacked structures [15]. Conversely, the addition of α-rice flour promotes starch swelling and gel formation, enhancing the viscoelasticity of the ink and mitigating structural deformation, thereby contributing to the maintenance of the printed structure’s shape after deposition [9].

Figure 3C displays the 3D-printed structures with varying sucrose concentrations, revealing that higher sucrose levels result in less stable forms. The deformation factor serves as an indicator of shape retention, closely related to the material’s mechanical properties [33]. Increasing α-rice flour and decreasing sucrose concentrations reduce structural deformation, yielding cuboid structures closer to the ideal form. This improvement is associated with enhanced self-supporting ability and increased stiffness, as lower sucrose content strengthens the structure’s ability to maintain its shape [34]. In contrast, samples with higher sucrose concentrations showed increased deformation factor and reduced structural stability after deposition, often forming trapezoidal shapes with expanded bottom areas compared to the top. These results suggest that increasing α-rice flour while reducing sucrose concentration enhances the viscoelasticity of the paste, improving printability and the structural integrity of stacked 3D-printed structures.

### 3.3. Correlations Between Printability and Rheology

**Figure 4:**
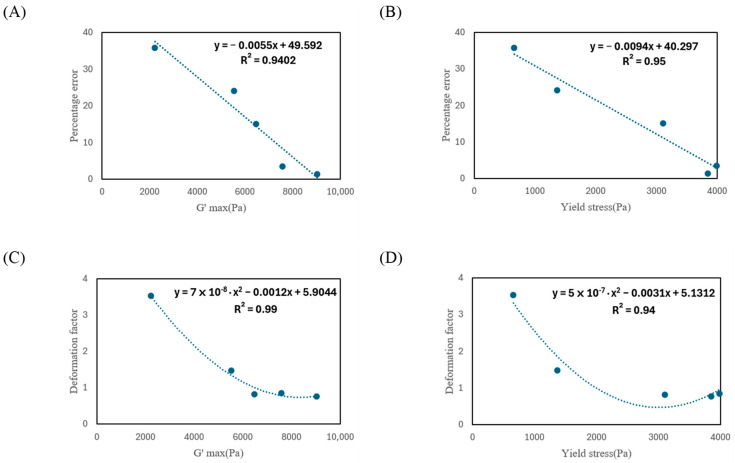
Correlation between rheological properties and 3D printability of α-rice flour–sucrose paste. (**A**) Plot of percentage error versus G′_Max_. (**B**) Plot of percentage error versus τy. (**C**) Plot of deformation factor versus G′_Max_. (**D**) Plot of deformation factor versus τy.

The correlation between the rheological properties and 3D printing printability of the α-rice flour–sucrose paste is presented in Figure 4. According to Zhu et al. [1], G′ is the only mechanical property that systematically changes with the stability of printed structures. Therefore, this study analyzed the correlation between G′_Max_, τy, and the printability of 3D-printed structures of the paste.

As shown in Figure 4A,B, G′_Max_ and τy exhibited negative correlations with the printing percentage error. This indicates that as G′_Max_ and τy increase, the printing percentage error decreases, suggesting that viscoelastic properties, influenced by the reduction in sucrose-induced water competition and the enhanced water availability for starch hydration, play a crucial role in improving printing accuracy. This negative correlation can be attributed to the fact that higher G′_Max_.and τy enhance the stiffness and structural stability of the material, increasing its resistance to external forces during printing and effectively suppressing deformation [35]. The coefficients of correlations (R^2^) between G′_Max_, τy, and the printing percentage error were 0.94 and 0.95, respectively, indicating strong correlations. These high R^2^ values suggest a strong relationship between the variables and that the regression model effectively explains the variability in the data.

A key implication of these findings is that materials with greater G′_Max_ and τy exhibit improved structural integrity throughout the extrusion and post-deposition stages [36]. This characteristic is especially crucial in 3D food printing, where maintaining the intended shape and functional attributes of the printed product is essential. A stronger and more stable material, as indicated by higher G′_Max_ and τy, offers better resistance to gravitational forces and external disturbances, ultimately enhancing shape retention and reducing deformation [6].

While increased G′_Max_ and τy generally enhance printing accuracy, overly rigid materials can create difficulties during extrusion [35]. If the material is too stiff, greater force is needed to push it through the nozzle, which may result in mechanical strain or uneven flow [37]. To ensure both precise printing and smooth extrusion, it is essential to strike a balance between rheological characteristics and material flowability. Figure 4C,D show the correlations between G′_Max_, τy, and the deformation factor. The correlation between G′_Max_ and the deformation factor was analyzed using a quadratic regression model, yielding an R^2^ of 0.99, indicating a very strong correlation. This suggests that as G′_Max_ increases, the deformation factor significantly decreases, highlighting the importance of high G′ in enhancing the structural stability of printed structures [4]. Similarly, the relationship between τy and the deformation coefficient was also analyzed using a quadratic model, with an R^2^ value of 0.94, indicating a strong correlation. This implies that as τy increases, the deformation factor tends to decrease, meaning that higher τy strengthens the structural stability of the material, effectively suppressing deformation in stacked structures [35].

It is interesting to note that, unlike the percentage error, the deformation factor exhibited a non-linear relationship with G′_Max_ and τy. The inclusion of a quadratic term in the regression model implies that changes in G′_Max_ and τy do not affect deformation in a simple proportional manner [38]. Instead, as G′_Max_ or τy increases, the rate at which the deformation factor decreases may accelerate or decelerate at different stages. This suggests a potential threshold effect, where beyond a certain point, increasing G′_Max_ or τy further providing diminishing returns in reducing deformation. This threshold effect plays a crucial role in regulating the viscoelastic properties of materials in 3D printing. When G′_Max_ and τy exceed the optimal range, extrusion imbalance and reduced interlayer adhesion may occur during the printing process, ultimately compromising the shape retention and dimensional accuracy of the final structure.”

From a 3D printing perspective, this quadratic relationship is particularly important in determining optimal rheological properties. While higher G′_Max_ and τy contribute to better shape retention and structural stability, excessive increases could lead to materials that are too rigid, which may introduce new challenges such as poor extrudability or nozzle clogging [37]. The quadratic trend may also indicate that at lower values, small increases in G′_Max_ or τy lead to significant improvements in structural integrity, whereas at higher values, the improvements plateau.

## 4. Conclusions

Sucrose disrupted the starch network, weakening viscoelastic properties, whereas α-rice flour promoted water absorption and gel formation, thereby enhancing printability and structural stability. Additionally, α-rice flour effectively improved viscoelastic properties and maintained structural integrity, demonstrating high potential for industrial applications in 3D food printing. This study quantitatively analyzed the impact of sucrose concentration on the rheological and printing properties of α-rice flour-based paste, providing a scientific basis for optimizing paste formulations for 3D printing. The results confirmed that G′_Max_ and τy significantly influence the shape retention and printing accuracy of printed structures, highlighting the strong correlation between viscoelastic properties and print stability. Furthermore, unlike previous studies, this research applied pregelatinized starch to 3D printing, demonstrating how starch pregelatinization enhances printability and structural stability. This approach expands the applicability of starch-based materials in 3D food printing and underscores the role of pregelatinized starch in improving print performance, distinguishing this study from prior research. However, this study considered sucrose as a single additive, without evaluating the synergistic effects of multiple food additives, which may further influence rheological behavior and printability. Moreover, time-dependent behaviors, such as thixotropic recovery, were not assessed, limiting a comprehensive evaluation of the long-term structural stability of printed constructs. Future studies should quantitatively evaluate the post-extrusion structural recovery of α-rice flour–sucrose paste by applying time-dependent flow behavior analysis and in-shear structural recovery measurements related to thixotropic recovery. These investigations will enable a deeper understanding of the viscoelastic recovery of the paste and facilitate the exploration of synergistic effects among food additives, such as salts, proteins, and hydrocolloids. Such findings will contribute to optimizing the formulation of 3D printing pastes for improved printability and structural stability.

Another future theme could include threshold values for G′_Max_ and τy, beyond which extrusion difficulties outweigh the benefits of improved shape retention. Additionally, investigating how temperature variations or the addition of biopolymers modulate the established correlations between rheological properties, printing accuracy, and structural stability could provide further insights into enhancing 3D printability and post-print stability.

## Figures and Tables

**Table 1:** Concentration levels of ingredients used for the preparation of α-rice flour–sucrose paste.

Ingredients	Mass (g/50 g)				
	S0	S1	S2	S3	S4
α-Rice flour	17.5	17	16.5	16	15.5
Sucrose	0	0.5	1	1.5	2
Distilled water	32.5	32.5	32.5	32.5	32.5
Total	50	50	50	50	50

**Table 2:** Rheological properties of α-rice flour–sucrose paste.

Formulation	Storage Modulus G′_Max_ (Pa)	Loss Modulus G″_Max_ (Pa)	Yield Stress τ_y_ (Pa)
S0	9032.0 ± 352.0 ^a (1) (2)^	5448.1 ± 360.3 ^a^	3845.3 ± 284.2 ^a^
S1	7574.0 ± 145.6 ^b^	5517.9 ± 383.6 ^a^	3986.0 ± 435.3 ^a^
S2	6468 ± 291.4 ^c^	4580.1 ± 175.5 ^b^	3104.7 ± 43.7 ^b^
S3	5529.5 ± 403.9 ^d^	1533.2 ± 148.1 ^c^	1364.7 ± 142.5 ^c^
S4	2212.1 ± 397.6 ^e^	739.1 ± 103.1 ^d^	658.8 ± 94.7 ^d^

(1) Mean ± standard deviation. (2) Means with different letters in the same column for each parameter are significantly different (*p* < 0.05).

## Data Availability

The data presented in this study are available on request from the corresponding author. The data are not publicly available due to privacy restrictions.

## Supplementary Materials

The following supporting information can be downloaded at: https://www.mdpi.com/article/10.3390/foods14071107/s1, Table S1: One-way ANOVA results for G′Max (storage modulus) of α-rice flour–sucrose pastes.; Table S2: One-way ANOVA results for G″Max (loss modulus) of α-rice flour–sucrose pastes.; Table S3: One-way ANOVA results for yield stress (τy) of α-rice flour–sucrose pastes.; Table S4: One-way ANOVA results for printing percentage error of 3D-printed hollow cylinders.; Table S5: One-way ANOVA results for deformation factor of 3D-printed cuboid samples.

## References

[1] Zhu S., Stieger M.A., van der Goot A.J., Schutyser M.A.I. (2019). Extrusion-based 3D printing of food pastes: Correlating rheological properties with printing behaviour. Innov. Food Sci. Emerg. Technol..

[2] Cheng Y., Yuqing H., Xiao L., Gao W., Kang X., Sui J., Cui B. (2024). Impact of starch amylose and amylopectin on the rheological and 3D printing properties of corn starch. Int. J. Biol. Macromol..

[3] Maldonado-Rosas R., Tejada-Ortigoza V., Cuan-Urquizo E., Mendoza-Cachú D., Morales-de la Peña M., Alvarado-Orozco J.M., Companella O.H. (2022). Evaluation of rheology and printability of 3D printing nutritious food with complex formulations. Addit. Manuf..

[4] Liu Z., Bhandari B., Parkash S., Mantibal S., Zhang M. (2019). Linking rheology and printability of a multicomponent gel system of carrageenan-xanthan-starch in extrusion based additive manufacturing. Food Hydrocoll..

[5] Solaesa A.G., Villanueva M., Vela A.J., Ronda F. (2022). Impact of microwave radiation on in vitro starch digestibility, structural and thermal properties of rice flour. From dry to wet treatments. Int. J. Biol. Macromol..

[6] Liu Y., Tang T., Duan S., Qin Z., Li C., Zhang Z., Liu A., Wu D., Chen H., Han G. (2020). Effects of sodium alginate and rice variety on the physicochemical characteristics and 3D printing feasibility of rice paste. LWT.

[7] Martínez-Cano A., Mendoza-Báez R., Zenteno-Mateo B., Rodríguez-Mora J.S., Agustín-Serrano R., Morales M.A. (2022). Study by DFT of the functionalization of amylose/amylopectin with glycerin monoacetate: Characterization by FTIR, electronic and adsorption properties. J. Mol. Struct..

[8] Tao X., Zhang Y., Chen F., Huang Y., Chen P. (2021). Effects of sucrose on pasting, thermal, rheological and textural properties of native and alcohol-alkali-treated waxy rice starch treatments. Int. J. Biol. Macromol..

[9] Ma H., Liu M., Liang Y., Zheng X., Sun L., Dang W., Li J., Li L., Liu C. (2022). Research progress on properties of pre-gelatinized starch and its application in wheat flour products. Grain Oil Sci. Technol..

[10] Park J., Kim Y.R. (2021). Clean label starch: Production, physicochemical characteristics, and industrial applications. Food Sci. Biotechnol..

[11] Li Q., Liu S., Obadi M., Jiang Y., Zhao F., Jiang S., Xu B. (2020). The impact of starch degradation induced by pre-gelatinization treatment on the quality of noodles. Food Chem..

[12] Wang H., Xiao N., Wang X., Zhao X., Zhang H. (2019). Effect of pregelatinized starch on the characteristics, microstructures, and quality attributes of glutinous rice flour and dumplings. Food Chem..

[13] He X.H., Xia W., Chen R.Y., Dai T.T., Luo S.J., Chen J., Liu C.M. (2020). A new pre-gelatinized starch preparing by gelatinization and spray drying of rice starch with hydrocolloids. Carbohydr. Polym..

[14] Donmez D., Pinho L., Patel B., Desam P., Campanella O.H. (2021). Characterization of starch–water interactions and their effects on two key functional properties: Starch gelatinization and retrogradation. Curr. Opin. Food Sci..

[15] Bak J., Yoo B. (2023). Rheological characteristics of concentrated ternary gum mixtures with xanthan gum, guar gum, and carboxymethyl cellulose: Effect of NaCl, sucrose, pH, and temperature. Int. J. Biol. Macromol..

[16] Kim S.M., Woo J.H., Kim H.W., Park H.J. (2022). Formulation and evaluation of thermoreversible sugar-paste for hot-melt 3D printing. J. Food Eng..

[17] Liu Z., Yang J., Shi Z., Chen L., Zheng B. (2021). Effect of stearic acid on the microstructural, rheological and 3D printing characteristics of rice starch. Int. J. Biol. Macromol..

[18] Barrios-Rodríguez Y.F., Igual M., Martínez-Monzó J., García-Segovia P. (2024). Exploration of changes in rheological and spectral properties of rice protein inks before and after 3D printing. LWT.

[19] Cheng Y., Liang K., Chen Y., Gao W., Kang X., Li T., Cui B. (2023). Effect of molecular structure changes during starch gelatinization on its rheological and 3D printing properties. Food Hydrocoll..

[20] Zheng L., Ren A., Liu R., Xing Y., Yu X., Jiang H. (2022). Effect of sodium chloride solution on quality of 3D-printed samples molded using wheat starch gel. Food Hydrocoll..

[21] Woodbury T., Grush E., Allan M.C., Mauer L.J. (2022). The effects of sugars and sugar alcohols on the pasting and granular swelling of wheat starch. Food Hydrocoll..

[22] Niu B., Chao C., Cai J., Yan Y., Copeland L., Wang S., Wang S. (2019). The effect of NaCl on the formation of starch-lipid complexes. Food Chem..

[23] Huang Y., Bao X., Li P., Zhan L., Wu H., Chen P. (2022). Effect of NaCl addition on alcohol-alkali-treated waxy rice starch: Structural and physicochemical functionality. Food Chem..

[24] Mohamed O.B. (2021). Effects of processing and additives on starch physicochemical and digestibility properties. Carbohydr. Polym. Technol. Appl..

[25] Gholamipour-Shirazi A., Norton I.T., Mills T. (2019). Designing hydrocolloid based food-ink formulations for extrusion 3D printing. Food Hydrocoll..

[26] Kim J.H., Chang Y.H., Lee Y.S. (2023). Effects of NaCl on the Physical Properties of Cornstarch–Methyl Cellulose Blend and on Its Gel Prepared with Rice Flour in a Model System. Foods.

[27] Fernández-Cervantes I., Morales M.A., Agustín-Serrano R., Cardenas-García M., Pérez-Luna P.V., Arroyo-Reyes B.L., Maldonado-García A. (2019). Polylactic acid/sodium alginate/hydroxyapatite composite scaffolds with trabecular tissue morphology designed by a bone remodeling model using 3D printing. J. Mater. Sci..

[28] Geffrault A., Bessaies-Bey H., Roussel N., Coussot P. (2023). Printing by yield stress fluid shaping. Addit. Manuf..

[29] Nijdam J.J., LeCorre-Bordes D., Delvart A., Schon B.S. (2021). A rheological test to assess the ability of food inks to form dimensionally stable 3D food structures. J. Food Eng..

[30] Zhou J., Meng X. (2024). Effect of glycerol incorporation on the liquid crystal structure of sucrose fatty acid ester in aqueous solution. Colloids Surf. A Physicochem. Eng. Asp..

[31] Sun B., Qian X., Cui G., Ma S., Wang X. (2023). Synergistic effect of combined sucrose substitutes and partially gelatinized oat flour on gluten-free steamed oat cakes produced only by oat flour. J. Cereal Sci..

[32] Outrequin T.C.R., Gamonpilas C., Siriwatwechakul W., Sreearunothai P. (2023). Extrusion-based 3D printing of food biopolymers: A highlight on the important rheological parameters to reach printability. J. Food Eng..

[33] Shen H., Yu J., Bai J., Liu Y., Ge X., Li W., Zheng J. (2023). A new pre-gelatinized starch preparing by spray drying and electron beam irradiation of oat starch. Food Chem..

[34] Ainis W.N., Feng R., van den Berg F.W.J., Ahrné L. (2023). Comparing the rheological and 3D printing behavior of pea and soy protein isolate pastes. Innov. Food Sci. Emerg. Technol..

[35] Chen H., Xie F., Chen L., Zheng B. (2019). Effect of rheological properties of potato, rice and corn starches on their hot-extrusion 3D printing behaviors. J. Food Eng..

[36] Tejada-Ortigoza V., Cuan-Urquizo E. (2022). Towards the development of 3D-printed food: A rheological and mechanical approach. Foods.

[37] Chen J., Zhang M., Mujumdar A.S., Phuhongunge P. (2022). 4D printing induced by microwave and ultrasound for mushroom mixtures: Efficient conversion of ergosterol into vitamin D2. Food Chem..

[38] James G., Witten D., Hastie T., Tibshirani R., Taylor R. (2023). An Introduction to Statistical Learning: With Applications in Python.

