# Supplementary material for: Effect of Sucrose on the Rheology and 3D Printability of Pregelatinized Rice Flour Paste
Source: Foods. 2025 Mar 23;14(7):1107. doi: 10.3390/foods14071107 (PMC11989006; doi:10.3390/foods14071107)
Supplement: Supplementary file 1 [file foods-14-01107-s001.zip › foods-3514604-supplementary.pdf]

Supplementary Table S1.

One-way ANOVA results for the storage modulus (G') of α-rice flour-sucrose paste with varying sucrose concentrations

Source	DF[1)]	Sum of squares	Mean squares	F[2)]	Pr > F[3)]	p-values signification codes[4)]
Model	4.0	79017260.0	19754315.0	179.2	**<0.0001**	***
Error	10.0	1102386.6	110238.7			
Corrected Total	14.0	80119646.6				

1)DF = Degrees of freedom.
2)F = F-statistic value.
3)Pr > F = Probability value for statistical significance.
4)p-values significance codes: $p < 0.001$ (***).

Supplementary Table S2.

One-way ANOVA results for the loss modulus (G") of α-rice flour-sucrose paste with varying sucrose concentrations

Source	DF[1)]	Sum of squares	Mean squares	F[2)]	Pr > F[3)]	p-values signification codes[4)]
Model	4.0	61513479.6	15378369.9	225.9	**<0.0001**	***
Error	10.0	680775.8	68077.6			
Corrected Total	14.0	62194255.3				

[1)]DF = Degrees of freedom.
[2)]F = F-statistic value.
[3)]Pr > F = Probability value for statistical significance.
[4)]p-values significance codes: $p < 0.001$ (***).

Supplementary Table S3.

One-way ANOVA results for the yield stress (τy) of α-rice flour-sucrose paste with varying sucrose concentrations

Source	DF[1)]	Sum of squares	Mean squares	F[2)]	Pr > F[3)]	p-values signification codes[4)]
Model	4.0	27061937.2	6765484.3	112.2	**<0.0001**	***
Error	10.0	602976.1	60297.6			
Corrected Total	14.0	27664913.3				

[1)]DF = Degrees of freedom.
[2)]F = F-statistic value.
[3)]Pr > F = Probability value for statistical significance.
[4)]p-values significance codes: $p < 0.001$ (***).

Supplementary Table S4.

One-way ANOVA results for the percentage error of 3D-printed α-rice flour-sucrose paste structures

Source	DF[1)]	Sum of squares	Mean squares	F[2)]	Pr > F[3)]	p-values signification codes[4)]
Model	4.0	2499.4	624.9	64.3	**<0.0001**	***
Error	10.0	97.2	9.7			
Corrected Total	14.0	2596.6				

[1)]DF = Degrees of freedom.
[2)]F = F-statistic value.
[3)]Pr > F = Probability value for statistical significance.
[4)]p-values significance codes: $p < 0.001$ (***).

Supplementary Table S5.

One-way ANOVA results for the deformation factor of 3D-printed α-rice flour-sucrose paste structures

Source	DF[1)]	Sum of squares	Mean squares	F[2)]	Pr > F[3)]	p-values signification codes[4)]
Model	4.0	16.6	4.1	36.4	**<0.0001**	***
Error	10.0	1.1	0.1			
Corrected Total	14.0	17.7				

[1)]DF = Degrees of freedom.
[2)]F = F-statistic value.
[3)]Pr > F = Probability value for statistical significance.
[4)]p-values significance codes: $p < 0.001$ (***).
